# Oral high-dose sertraline-induced acute pancreatitis: a case report and literature review

**DOI:** 10.3389/fmed.2025.1613583

**Published:** 2025-07-21

**Authors:** Xiaoying Wu, Liwei Duan, Linhao Ma

**Affiliations:** ^1^Department of Emergency Medicine, Shanghai Fourth People's Hospital, School of Medicine, Tongji University, Shanghai, China; ^2^Department of Medical Affairs, Shanghai Fourth People’s Hospital, School of Medicine, Tongji University, Shanghai, China

**Keywords:** sertraline, acute pancreatitis, drug-induced pancreatitis, overdose, SSRIs

## Abstract

Acute pancreatitis (AP) is characterized by acute inflammation and pancreatic injury, with gallstones and chronic alcohol use representing the most common etiologies. Although drug-induced pancreatitis (DIP) accounts for fewer than 3% of AP cases, its recognition as a significant contributor to AP is growing. Sertraline, a widely prescribed selective serotonin reuptake inhibitor (SSRI), is associated with diverse adverse effects, even at therapeutic doses. We present a case of a 27-year-old female with a history of depression who developed mild acute pancreatitis following a sertraline overdose. Diagnostic evaluation, including computed tomography (CT) and serological analysis, revealed pancreatic parenchymal swelling and elevated serum amylase levels, confirming AP. Other potential causes were systematically excluded. The patient’s condition resolved following drug discontinuation and standard supportive therapy. This case underscores the need for heightened clinical awareness of SSRI-associated pancreatitis, particularly in the context of overdose.

## Introduction

Drug-induced pancreatitis (DIP) is a well-defined but underrecognized etiology of acute pancreatitis (AP), triggered by direct toxicity or metabolic effects of medications (1). Diagnosis requires fulfillment of established AP criteria (e.g., revised Atlanta classification), exclusion of alternative causes (e.g., gallstones, alcohol), and symptom resolution upon drug cessation. Despite its inclusion in clinical guidelines, DIP remains underdiagnosed due to nonspecific presentation, the absence of definitive biomarkers, and limited clinician familiarity with drug-related pancreatic injury—particularly for medications not classically associated with pancreatic toxicity (e.g., antidepressants, immunosuppressants) (2). More than 264 different drugs from various classes were found associated with AP, but as a result of the general lack of formal epidemiological studies, the magnitude of the risk of most of these medications remained unknown. Several studies have been published that have analysed how many cases of AP can be associated with the use of drugs (3). These studies have established the prevalence of drug-induced AP cases at 0.03% in Canada, 0.05% in Korea, 0.2% in France and 0.3% in Switzerland (4). However, mechanistic understanding remains incomplete, and epidemiological data are scarce due to underreporting and diagnostic challenges. This case report highlights sertraline, a selective serotonin reuptake inhibitor (SSRI), as a potential culprit in DIP—a association infrequently documented in the literature. We present a confirmed case of sertraline-induced AP following overdose, emphasizing the importance of pharmacovigilance and systematic exclusion of DIP in atypical AP presentations.

### Case description

**Figure 1:**
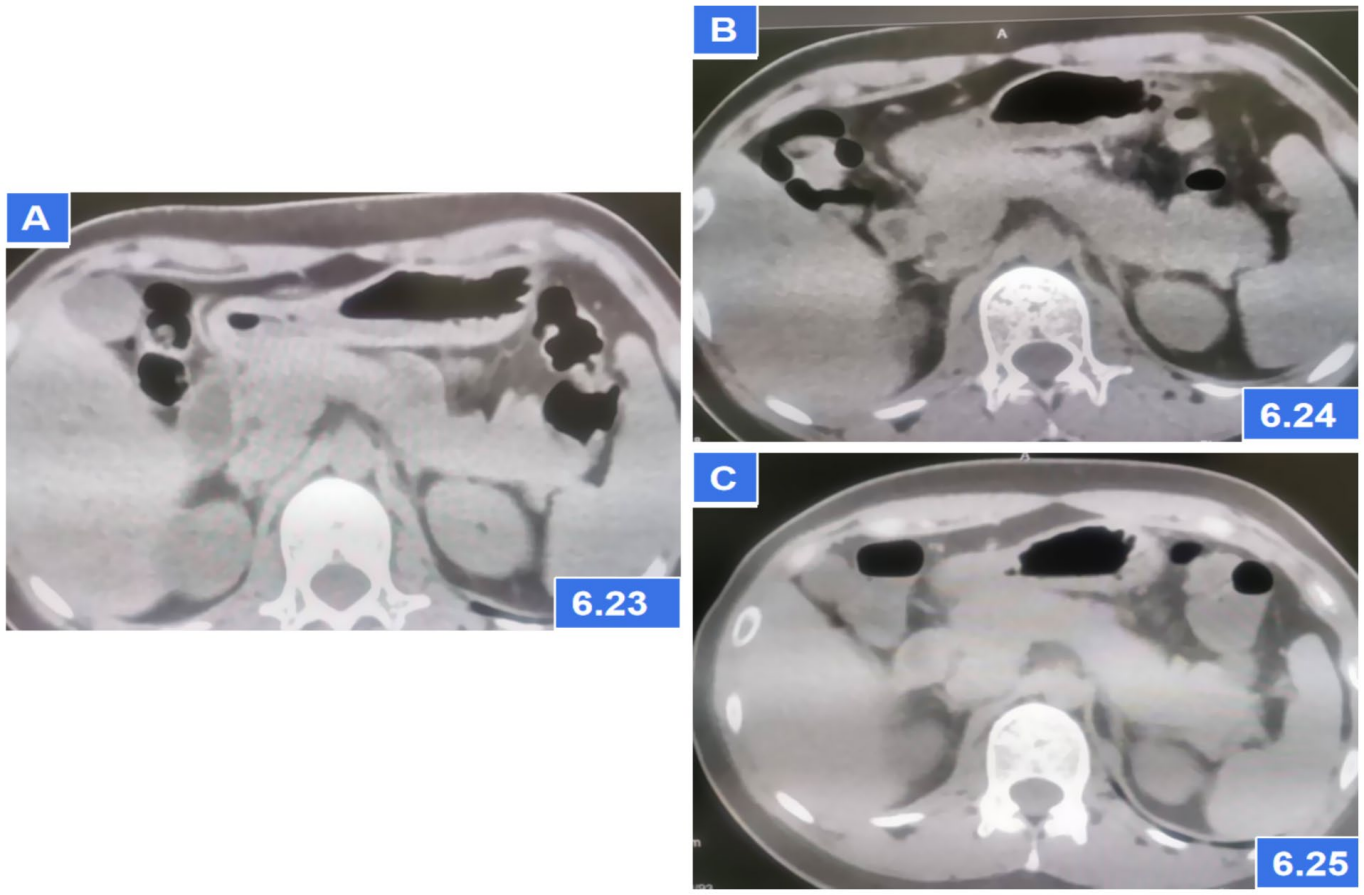
Abdominal CT showing pancreatic parenchymal swelling; **(A)** CT scans on the first day after admission; **(B)** CT scans at 6 h later after admission; **(C)** CT scans after AP diagnosis and treatment for 2 days.

A 27-year-old female presented to the emergency department 3 h after ingesting 1,000 mg of sertraline (20*50 mg tablets). She had been prescribed sertraline 75 mg daily for depression diagnosed 1 month prior. The patient denied alcohol use, binge eating, biliary disease, or prior pancreatic/surgical interventions (e.g., endoscopic retrograde cholangiopancreatography). On examination, she was ambulatory, afebrile (37.2°C), with stable vital signs (pulse 92 bpm, respiratory rate 20/min, BP 118/83 mmHg). Abdominal examination revealed no tenderness, rebound, or guarding. Cardiac and pulmonary auscultation was unremarkable. Laboratory findings at admission included: Serum amylase: 228 U/L (reference: 30–110 U/L); Leukocytosis: 13.23 × 10 < sup > 9</sup>/L; Total bilirubin: 24.4 μmol/L; Arterial blood gas: pH 7.43, actual bicarbonate 19 mmol/L, carbon dioxide partial pressure 3.9 KPa, lactate 3.2 mmol/L. Other parameters (liver enzymes, electrolytes) were within normal limits. Admission CT: No pancreatic or biliary abnormalities (Figure 1A). Twelve hours post-admission, serum amylase rose to 684 U/L, while repeat CT demonstrated pancreatic edema without biliary obstruction or gallstones (Figure 1B). Urinary amylase remained negative (inconsistent with serum trends; assay-specific limitations noted), aligning with atypical DIP presentations where urinary markers may lag behind serum elevations.

**Figure 2:**
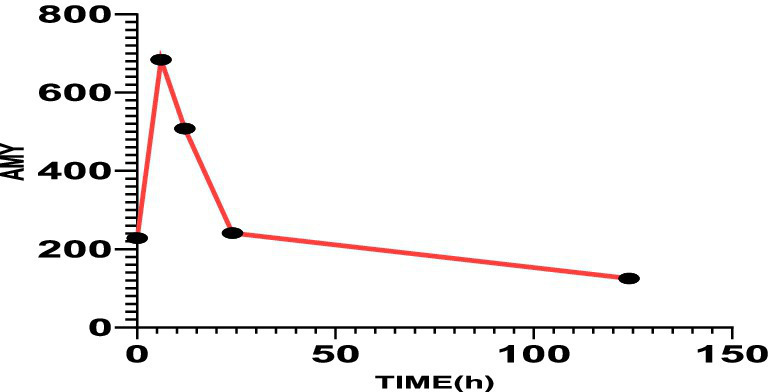
The serum amylase value on the first day after admission was 228 U/L, 6 h later was 684 U/L, 12 h later was 508 U/L, 24 h later was 241 U/L, and 120 h later was 125 U/L.

**Figure 3:**
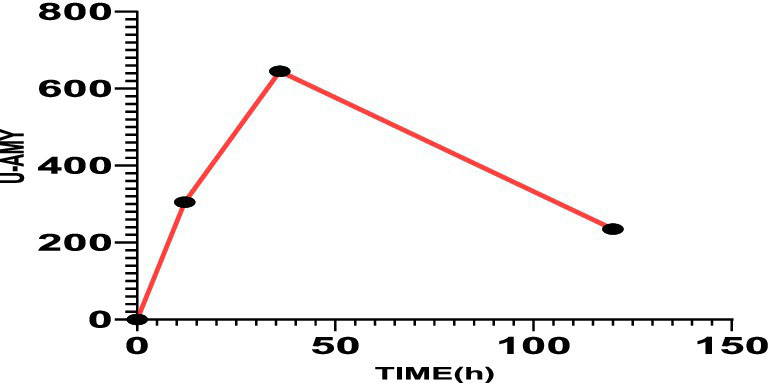
The value of urinary amylase was 305 U/L after 12 h, 645 U/L after 30 h, and 235 U/L after 120 h.

The patient met diagnostic criteria for AP per Chinese Guidelines (2021) (5) and Atlanta classification: (1) Serum amylase >3*upper limit; (2) Characteristic CT findings. Her BISAP score of 0 predicted mild disease. Management included: Gastric lavage (for overdose); fluid resuscitation (lactated Ringer’s: 250 mL/h), antibiotics (ceftriaxone), acid suppression and gastric mucosa protection (pantoprazole 40 mg IV BID); Supportive care (fasting). Monitoring: Serial amylase (down-trending to 241 U/L at 24 h) and repeat CT (normalization, Figure 1C). Sertraline was discontinued, and AP resolved without complications. On the third day of hospitalization (72 h), the patient had no complaints and requested discharge. Rechallenge with 50 mg sertraline during follow-up did not recur symptoms, suggesting dose-dependent toxicity. Combining the medical history and medication use, Per the China National Center for Adverse Drug Reaction Monitoring criteria (6), this case was graded as “probable” due to: Temporal association (onset ≤12 h post-overdose); Exclusion of alternative causes (biliary, alcohol, metabolic); Biochemical/histologic plausibility (SSRI-induced sphincter of Oddi dysfunction). Notably, rechallenge with sertraline 50 mg during follow-up did not recur, suggesting dose-dependent toxicity (Figures 2, 3).

## Discussion

Drug-induced pancreatitis (DIP) accounts for 0.1–3.4% of acute pancreatitis (AP) cases, with a rising incidence reported in recent years, primarily documented through case reports and retrospective studies. Due to ethical constraints, prospective studies or rechallenge trials are rarely feasible, leaving retrospective analyses and case series as the primary sources of evidence. Diagnosing DIP remains challenging, as definitive confirmation often requires drug rechallenge, which is seldom performed in clinical practice. Instead, most cases rely on presumptive diagnosis, based on temporal drug exposure and the exclusion of other etiologies. The lack of specific biomarkers or imaging features further complicates differentiation from other causes of AP. Currently, no universally accepted diagnostic criteria exist, and DIP remains largely a diagnosis of exclusion (7). A 2023 multicenter analysis of 1,060 DIP cases established antitumor agents (16.89%), antibiotics (12.08%), anticonvulsants (9.72%), and antipsychotics (3.77%) as the predominant causative agents, with severity stratification showing mild (68.77%), moderate (11.13%), and severe AP (20.09%) (8). Mechanistic studies demonstrate three validated pathways for DIP: (i) direct acinar cell toxicity via intracellular metabolite accumulation, (ii) sphincter of Oddi dyskinesia observed in cholescintigraphy studies, and (iii) delayed-type hypersensitivity reactions confirmed through lymphocyte transformation testing (9). While SSRIs account for <1% of DIP cases in pharmacovigilance databases, the temporal association in this case meets the Karch-Lasagna criteria for adverse drug reaction causality: (1) Chronological plausibility: Pancreatitis onset occurred 30 days after sertraline initiation, consistent with the 7–21 day latency period reported in confirmed SSRI-induced AP cases. (2) Dechallenge response: Serum lipase normalized (from 684 U/L to 125 U/L) within120 hours of discontinuation, mirroring the 72-h resolution window documented in prior cases. (3) Exclusion of confounders: Comprehensive workup ruled out gallstones (abdominal CT), hypertriglyceridemia (Due to the limited emergency conditions, blood lipid was not checked. However, after communication with the laboratory department, no lipemia was found in serum specimens, so hyperlipidemia was excluded) and alcohol use (4) Rechallenge data: The patient denied the history of pancreatitis, cholecystitis, gallstones and other drug history. He had an annual physical examination and no obvious gallbladder or pancreatic disease was found, suggesting a sertraline-specific effect. It has been found that after an overdose of sertraline or other selective serotonin reuptake inhibitors (SSRIs), some patients show no clinical symptoms, while others may present with somnolence, tremors, nausea, vomiting, dilated pupils, tachycardia, electrocardiogram changes, etc. (10). However, This case suggests that it may cause AP, providing partial support for sertraline-related AP. Combining the patient’s medication history, the temporal rationality of AP occurrence, the potential risk of sertraline-induced AP, improvement after discontinuation of the drug and symptomatic treatment, and other factors that may induce AP, a comprehensive analysis and judgment suggest that this may be DIP caused by sertraline. This biochemical and temporal profile aligns with the 2024 AGA guidelines for DIP diagnosis, while the rapid clinical improvement is consistent with a meta-analysis of a case–control study on the risk of acute pancreatitis and SSRI use (11). The Naranjo Adverse Drug Reaction Probability Scale score of 6 (“probable”) further supports this association.

The relationship between SSRIs and DIP remains controversial. A population-based case–control study including 4,631 AP patients found a possible association between SSRIs and AP (12). Another meta-analysis involving 17,548 AP patients from four studies showed that the combined odds ratio (OR) for AP risk with SSRIs was 1.26 (95% CI: 1.13–1.40). Subgroup analysis indicated that the first 2 weeks of SSRI use were a high-risk period for AP, with the risk 1.48 times higher than after 2 weeks. This meta-analysis provided evidence of a significant positive correlation between SSRI use and AP risk, with a higher risk within the first 2 weeks of use, which should be taken seriously (13).

Sertraline is one of the most widely used SSRIs and a classic antidepressant. However, due to inconsistent latency periods and the absence of rechallenge data—often precluded for ethical reasons—the causal relationship remains uncertain, sertraline is classified as Category IV according to the Badalov classification criteria. There are very few reports of sertraline causing DIP. We searched and collected published data on sertraline-induced DIP in PubMed and found only four cases to date. However, due to the lack of a consistent latency period and absence of drug rechallenge, the evidence is weak, and the classification has been updated to Category III (14). Recent studies emphasize the need for further pharmacovigilance to clarify this potential association, particularly as SSRIs continue to be extensively prescribed worldwide.

In this case, the patient exhibited no significant clinical symptoms 3 h after an acute sertraline overdose. Diagnosis of acute pancreatitis (AP) was confirmed biochemically (amylase elevation >3*the upper limit of normal) and radiologically (characteristic CT findings). Other etiologies were ruled out, as the patient denied alcohol use, comorbidities, or concurrent medications. Notably, the rapid onset following overdose and resolution upon drug discontinuation support sertraline as the likely culprit. Unlike prior reports implicating polypharmacy or alcohol, this case had minimal confounders. The patient had tolerated a therapeutic dose (50 mg/day) for 1 month without incident, suggesting dose-dependent toxicity, as AP occurred only after overdose and did not recur upon resuming the standard dose. While rechallenge was ethically unfeasible, the temporal association aligns with recent evidence linking SSRIs to drug-induced pancreatitis (DIP). This underscores the need for vigilance regarding AP in intentional overdoses, particularly with psychotropic agents.

## Conclusion

Drug-induced pancreatitis (DIP) is a rare etiology of acute pancreatitis (AP), associated with hundreds of medications. Familiarity with published case reports and research on implicated drugs is essential for clinicians to promptly recognize DIP and incorporate this possibility into therapeutic decision-making. This case report contributes clinical evidence supporting the association between sertraline and AP. Given the expanding use of antidepressants across diverse age groups, clinicians should maintain a high index of suspicion for SSRI-related acute pancreatitis, including sertraline, particularly in cases of intentional overdose. These findings underscore the importance of heightened clinical awareness and implement appropriate monitoring protocols for potential sertraline-induced acute pancreatitis. Especially in patients presenting after suicide attempts involving drug overdose.

## Data Availability

The original contributions presented in the study are included in the article/supplementary material, further inquiries can be directed to the corresponding author.
